# Clinical efficacy of *Psidium guajava* — based Herbal gel in the management of recurrent aphthous stomatitis: A randomized clinical trial

**DOI:** 10.12669/pjms.41.10.12445

**Published:** 2025-10

**Authors:** Emaan Mansoor, Ezza Mansoor, Efrah Mansoor, Afsheen Mansoor

**Affiliations:** 1 Emaan Mansoor, Islamic International Dental College, Riphah International University, Islamabad, Pakistan; 2 Ezza Mansoor, Islamic International Dental College, Riphah International University, Islamabad, Pakistan; 3 Efrah Mansoor, Islamic International Dental College, Riphah International University, Islamabad, Pakistan; 4 Afsheen Mansoor Associate Professor, Dental Material Sciences Department, School of Dentistry, Shaheed Zulfiqar Ali Bhutto Medical University, Islamabad, Pakistan. Department of Microbiology, Quaid-i-Azam University, Islamabad, Pakistan

**Keywords:** Analgesic, Aphthous Stomatitis (RAS), Guava Herbal gel, Oral ulcer, Remedy

## Abstract

**Objective:**

To investigate clinical efficacy of Psidium guajava-based herbal gel in reducing size of recurrent aphthous stomatitis (RAS) of patients eventually relieving pain associated with it.

**Methodology:**

Single-blinded, parallel group randomized clinical study which was conducted at School of Dentistry Shaheed Zulfiqar Ali Bhutto Medical University from 15^th^ June 2023 to 16^th^ January 2024. Total 204 participants were randomly assigned to Control and Experimental Groups where Control group was not given any treatment for RAS while Experimental group was advised to apply Herbal gel on ulcer 2-4 times/day for four weeks. Size of ulcer was assessed on first, second, third, fourth, seventh and fifteenth visit days for reduction and eventual healing. One-way ANOVA and POST HOC Tukey test was used to analyze data.

**Results:**

Baseline mean size of oral ulcer in both groups was 4.7 (±0.56) units. Experimental group exhibited greater mean ulcer size declination than Control group on every visit. On the second day of treatment, mean ulcer size measurement in Control group was 4.63 ± 0.17-mm and in Experimental group it was 4.29 ± 0.16-mm. On third and fourth days, Control group displayed mean ulcer size reduction of 3.95 ± 0.13-mm and 3.71 ± 0.11-mm as compared to 1.01± 0.07 cm² and 0.17 ± 0.05-mm in Experimental group. On the seventh day, mean oral ulcer size decreased to 2.46 ± 0.09-mm in Control group and disappeared in Experimental group (p = 0.001).

**Conclusion:**

Current study found that naturally procured herbal gel reduces size of recurrent aphthous stomatitis quickly confirming that oral ulcer can be treated with herbal gel more effectively that might be helpful in relieving pain associated with it.

## INTRODUCTION

Managing Recurrent Aphthous Stomatitis (RAS), the most frequent oral mucosal condition, is quite challenging in this epic.^1^,^2^ The global population experiences Recurrent Aphthous Stomatitis (oral ulcers) more oftenly on the greater extent.^3^ Patients between 10 and 40 years of age commonly experience “RAS” and it necessitates prompt treatment to prevent pain and discomfort associated with oral ulcers.^4^-^6^ Poor oral hygiene, traumatic cheek bites, localized oral trauma, hard brushing habits, vitamin deficiency, hormonal imbalance, microbial infection, oral pathologies, stress-induced lesions, food allergies and socioeconomic status collectively result in the development of “RAS” ulcers.^4^,^7^,^8^

Ulcers are recognized with well-defined red borders and a yellowish-gray pseudo-membranous center respectively. There are three main categories of “RAS” ulcers such as Minor, Major and Herpetiform where minor are considered as the most common ulcers. On developing such minor ulcer, an individual experiences a burning sensation and pricking pain in the oral mucosa within 24-48 hours. The size of such ulcer is less than 1-cm where self-limiting resolutions with no scarring occurs within more than seven to 10 days. Major aphthous stomatitis is identified by deep ulcers in the mouth having size greater than 1-cm. These Ulcers leave scars and take over a month to heal fully. Primarily, these ulcers arise during puberty and are characterized by multiple recurrences at every stage of life.^9^ Herpetiform ulcers exist in numerous small-sized lesions with multiple branches. These ulcers heal within a month without leaving any scar.^10^ The highlighted problem with ulcers is severe pain and discomfort associated with their size that needs attention for a healthy living and stress-free working of any individual. Other factors including Perceptual abilities, age, personal experiences, gender, occupation, social, cultural, and ethnic variables all play a role in intensifying the pain caused by ulcers. The main goal in managing recurrent aphthous stomatitis is shrinking the ulcer size for alleviating the pain associated with it in order to minimize the stress of eating and speaking.

The reduction in “RAS” ulcer sizes with associated pain may be achieved by utilizing different useful oral medicaments and gels. Sometimes patients may choose either self-treatment or seek out alternative therapies for treating the affected oral mucosa.^10^ A variety of immuno-modulatory, anti-inflammatory, and chlorhexidine-based topical drugs, including systemic antibacterial medicaments, are used for managing infections, but they may not be effective for every case.^2^,^11^,^12^ The efficacy of immunosuppressants, antihistamines, non-steroidal anti-inflammatory medications, local anesthetics, gamma-globulins, and enzymatic preparations as topical agents for therapeutic use is uncertain due to the insufficient number of well-designed controlled clinical trials.^13^,^14^ These patients frequently use other OTC products besides those specifically for ulcers, with uncertain benefits.

Currently, naturally procured oral health products from green plants became famous because of their safe production and biocompatible composition. A guava-extract based Denpro oral gel by Gennec Health Sciences PVT, LTD in Karachi, Pakistan, boasts advanced adhesive and protective properties, potentially expediting ulcer healing. The adhesive properties of Denpro oral gel come from its Carboxy methylcellulose constituent, which might form a potent, elastic and thin cushion against “RAS” ulcers, providing a durable and protective barrier for them. This topical agent’s herbal component, a 2% guava extract, can enhance the healing process with immediate pain relief at an ulcer site due to its medicinal properties. Limited data exist in the literature on the use of guava extract, in the form of an oral gel, for treating “RAS” ulcers. This study aimed to evaluate the clinical effectiveness of a herbal based commercial gel containing guava extract for reducing the size of oral ulcers in patients that might be helpful in diminishing the pain associated with them.

## METHODOLOGY

This study was designed as a single-blind, parallel-group randomized controlled trial with a 1:1 allocation ratio. The study was carried out at Restorative Department, School of Dentistry, Shaheed Zulfiqar Ali Bhutto Medical University, Islamabad between 15th June 2023 to 16th January 2024, lasting six months.

### Ethical Approval:

The Ethical Review Committee of the Dental hospital granted ethical approval (SOD/ERB/2023/43-01; Dated: June 12, 2023) and the trial was registered at US registry of ClinicalTrials.gov PRS with the identifier NCT06490289; Dated: July 5^th^, 2024.

### Patient selection, allocation and blinding:

The participants signed up for the test following a succinct account of the study’s goals. Their enrollment followed the approval of their written informed consents. They approved the publication of trial results as well. For this study, a non-probability consecutive sampling method was used. The Lottery method was used to assign participants randomly to two groups. The patients were grouped as Control (No treatment) and Experimental (Herbal based oral gel). In this trial, only the data analyst doctor who was designated for measuring the ulcer size remained unaware of the study groups due to its single-blind design. Data analyst doctor never knew about the Control group and Experimental group participants involved in the study to avoid any bias.

### Sample size calculation:

Initially 213 participants were assessed for eligibility of which 09 were dropped out due to various reasons before the commencement of this clinical trial. The sample size constituted of 204 participants with a=0.05, 80% power and d=0.40.^15^ Total 204 participants attended the entire follow up of this interventional study. Fifty-five males and 149 females took part in this clinical trial.

### Inclusion criteria:

Mouth ulcer size greater than 2-mm that appeared within 48 hours. Young patients ranging between 18-25 years of age were enrolled in this study where stress and food triggers were more common in Pakistan.^16^ All healthy participants with no history of infectious, haematological, gastrointestinal or dermatological disease that might give rise to oral mucosal ulceration. These participants agreed not to use any topical, systemic analgesics or anti-inflammatory medicines including ibuprofen, paracetamol, corticosteroids, aspirin, alcoholic mouthwashes, corticosteroids and tooth bleaching agents.^15^

### Exclusion criteria:

Individuals having any past allergy history for herbal gels. Individuals who have undergone any invasive dental procedures two weeks prior screening. Individuals with uncontrolled chronic disease history such as chronic liver disease, chronic renal disease. Individuals who have currently used any medication for ulcer treatment and developed a recurrent ulcer within a month. The female patients with positive pregnancy tests were also excluded from this study.^15^

In the Control group, no treatment for ulcer management was given, while the Experimental group received treatments using a guava-extract based herbal. Small amount of this gel was taken on fingertip/applicator and applied topically in a swiping action on the ulcer two to four times daily for at least four weeks. After meals or before sleeping, patients should apply the gel without using any additional topical or systemic medications for ulcer treatment.

### Assessment of oral ulcer size:

The ulcer size was measured at baseline i.e Visit-1 (Day 0) and then at subsequent visits: Visit-2 (Day-1), Visit-3 (Day-4), Visit-4 (Day-7) and Visit-5 (Day-14). The recurrent aphthous stomatitis was checked for size reduction throughout the multiple visits.

### Statistical analysis:

Mean, standard deviation and standard error for the data were computed with One way ANOVA in SPSS version 26 (IBM-Corporation, Armonk, New York, USA). The significance of the difference between visits in our study, as assessed by Post Hoc Tukey test, was kept below 0.05.

## RESULTS

**Fig.1:**
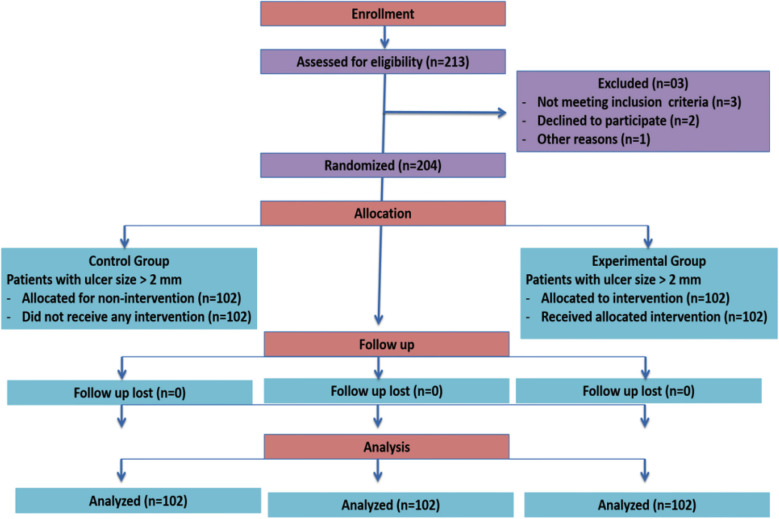
CONSORT 2010 Flow Diagram.

In the Control group, there were 34 male and 69 female patients among the 204 participants with mouth ulcers larger than 2-mm, while 21 males and 80 females were assigned to the Experimental group. The Experimental group received Herbal oral gel for mouth ulcer treatment, while the Control group did not receive any treatment. The CONSORT 2010 Flow diagram (Fig.1) details this.

Patients ranged between 18- 25 years of age were enrolled in this study. The means of ages for males in this case were 20.16 years old with a standard deviation of 5.97, while females’ means were 21.21 years old with a standard deviation of 4.84. The baseline means size of mouth ulcers, identical in the Control and Experimental groups, was 4.77± 0.56-mm at Visit-1 (Day-0) (p =1.00). In both groups, the size of mouth ulcers showed a systematic decline, yet the decline was more prominent in the Experimental group.

The linear pattern reduction in the mean size of ulcer was observed in both the control and experimental groups after application of herbal gel. This reduction in mean ulcer size was measured on subsequent visits: Visit-1 (Day-0), Visit-2 (Day-1), Visit-3 (Day-4), Visit-4 (Day-7) and Visit-5 (Day-14). The mean size of mouth ulcer got reduced to 4.63 ± 0.17-mm in the Control group participants and 4.29 ± 0.16 -mm in the Experimental group participants at their Visit-2 (Day-1). The mean ulcer size declined to 3.95± 0.13-mm and 3.71± 0.11-mm in the Control group participants at their Visit-3 (Day-4) and Visit-4 (Day-7) whereas mean ulcer size reduced to 1.01 ± 0.07-mm and 0.17 ± 0.05 on the corresponding days with regular application of the gel. The last Visit-5 (Day-14) of Experimental group participants revealed complete recovery of ulcer by bringing its size to 0.00± 0.00-mm as compared to the Control group participants where it became 2.46 ± 0.09-mm (p = 0.001) (Table-I). The ANOVA test revealed statistically significant declines in mean mouth ulcer size among Control and Experimental groups at various follow up days (p = 0.001). Table-II reveals the rapid and comprehensive decrease in mean mouth ulcer size through Post Hoc Tukey analysis comparing the inter-group differences in the reduction of mean ulcer size at multiple visits (p < 0.05).

**Table-I:** One way ANOVA test comparing mean mouth ulcer size reduction between Control group and Experimental group participants at different follow-up visits.

Mean Size of Ulcer	Control Group using No Oral Gel	Experimental Group using Denpro Oral Gel	P-Value
Visit-1 (Day-0)	4.77± 0.56	4.77± 0.56	1.000
Visit-2 (Day-1)	4.63± 0.17	4.29± 0.16	0.001
Visit-3 (Day-4)	3.95± 0.13	1.01± 0.07	
Visit-4 (Day-7 )	2.71± 0.11	0.73± 0.05	
Visit-5 (Day-14)	1.46± 0.09	0.00± 0.00	

## DISCUSSION

Current study concluded that Herbal based guava extract gel healed the oral ulcer more quickly and effectively on the 7^th^ day (Visit=4). This amplified quick recovery of mean ulcer size is the strength of this study eventually becoming a trademark that might be helpful in relieving the excessive pain caused by it. The findings of this study are important and clinically relevant because it surpassed the prior literature due to the use of a guava extract-oriented gel versus prior mouthwashes.^3^ Our trial suggests that the oral ulcers might have healed more rapidly due to the enhanced adhesion properties of the herbal gels that might have been possibly due to the available natural components in their compositions. That might have created a strong and protective coating on the ulcer surface in turn encapsulating the infective tissue. This purely bonded encapsulation could be solely responsible for the effective pain relief and quick healing of oral ulcers in this study.

Over the past few decades, the use of latest biologically synthesized products utilizing plants, herbs and microbes have experienced remarkable growth in both developing and developed regions as the primary treatment modality in complementary and alternative medicine.^17^-^19^ The natural compounds in plants could potentially yield beneficial results when utilized in medicines, oral drugs, oral hygiene products and oral gels. This study assessed the effectiveness of a guava extract oral gel in relieving the pain of oral ulcers. The seventh day “Visit=4” of this study was earlier than in other past researches for ulcer recovery, as shown in Table-II.^3^ Researchers have found that “RAS” ulcers may heal on their own within 10-14 days,^15^ but patients may experience significant pain during this period some researchers have examined the impact of specific palliative methods on the ulcers’ healing time and pain relief.

**Table-II:** Post Hoc Tukey analysis comparing inter-group differences in ulcer size reduction at multiple visits.

Control group at different number of Visits	Comparison of mean Ulcer Size in Experimental group at different number of visits	Mean Difference with Standard Error (S.E)	p-value
Control group Visit-1(Day-0)	Experimental group Visit-1(Day-0)	0.00 ± 0.03	1.000
	Experimental group Visit-2(Day-1)	0.48 ± 0.03	0.002
	Experimental group Visit-3(Day-4)	3. 76 ± 0.03	0.004
	Experimental group Visit-4(Day-7)	4.6 ± 0.03	0.004
	Experimental group Visit-5(Day-14)	4.77 ± 0.03	0.001
Control group Visit-2(Day-1)	Experimental group Visit-1(Day-0)	- 0.13 ± 0.03	0.003
	Experimental group Visit-2(Day-1)	0.34 ± 0.03	0.009
	Experimental group Visit-3(Day-4)	3.63 ± 0.03	0.001
	Experimental group Visit-4(Day-7)	4.47 ± 0.03	0.007
	Experimental group Visit-5(Day-14)	4.63 ± 0.03	0.004
Control group Visit-3(Day-4)	Experimental group Visit-1(Day-0)	-0.58 ± 0.03	0.005
	Experimental group Visit-2(Day-1)	-0.10 ± 0.03	0.004
	Experimental group Visit-3(Day-4)	3.17 ± 0.03	0.003
	Experimental group Visit-4(Day-7)	4.02 ± 0.03	0.004
	Experimental group Visit-5(Day-14)	4.19 ± 0.03	0.008
Control group Visit-4(Day-7)	Experimental group Visit-1(Day-0)	-2.61 ± 0.03	0.003
	Experimental group Visit-2(Day-1)	-2.14 ± 0.03	0.006
	Experimental group Visit-3(Day-4)	1.14 ± 0.03	0.002
	Experimental group Visit-4(Day-7)	1.98 ± 0.03	0.004
	Experimental group Visit-5(Day-14)	2.15 ± 0.03	0.009
Control group Visit-5(Day-14)	Experimental group Visit-1(Day-0)	-3.31 ± 0.03	0.007
	Experimental group Visit-2(Day-1)	-2.83 ± 0.03	0.001
	Experimental group Visit-3(Day-4)	0.45 ± 0.03	0.003
	Experimental group Visit-4(Day-7)	1.29 ± 0.03	0.003
	Experimental group Visit-5(Day-14)	1.46 ± 0.03	0.005

The studies have showed ulcer reduction with associated pain slowly than in the current trial, yet symptoms resolved a day earlier, on the seventh day (Visit 4) instead of the tenth and fourteenth days (Visits 5 and 6).^20^ Some studies reported that commercially available gel without herbal ingredients reduced pain and size of “RAS” ulcers in about 30-50%^15^ of cases and 36.0% of cases,^21^ while other studies only showed a reduction in pain.^21^ These ulcers negatively affect the quality of life by hindering speech, mastication and swallowing.^22^,^23^ According to the present study, the use of herbal-based gel led to a 100% decrease in ulcer size that might have reduced the pain compared to past studies, suggesting an improvement in quality of life. In this clinical trial, the fast and effective relief from pain and reduction in ulcer size after applying the herbal based oral gel can be attributed to the main natural components including *Psidium guajava*.

Numerous herbal plants exhibit analgesic and anti-inflammatory properties due to a range of natural components like oleanolic acid, ursolic acid, uvaol, carotenoids, alkaloids, quercetin, saponins, tannins, guiiajaverin, polyphenolic compounds, ellagic acid, amritoside, triterpenoids, flavonoids, leucocyanidin, beta-sitosterol and other phytochemicals present in them.^24^ The significant anti-inflammatory property of Psidium guajava (guava) can be attributed to its rich content of flavonoids and other extracts, which primarily impede the production of protein kinases. These regulatory enzymes significantly contribute to inflammation and other immunologic processes.^24^ The precise quantities of flavonoids, carboxymethyl cellulose, glycerol, cetylpyridium chloride and menthahaplocalyx in the oral gel used in this trial contributed to the rapid healing of mouth ulcers.

Certain studies have shown that guava (Psidium guajava) has a wide range of pharmacological activities and is used in medical and dental treatments but not limited to hypertension, diabetes, dysentery, wound management, gastroenteritis, fever, vaginal problems, cough, cancer, dental caries, gingivitis, periodontitis and oral ulcers.^25^,^26^ Guava’s properties include analgesic, anti-hyperglycemic, anti-allergic, anti-tumor, anti-mutagenic, anti-inflammatory, anti-parasitic and anti-bacterial effects.^18^ Mouthwashes containing guava have been traditionally used for treating mouth ulcers,^27^ especially in the developing regions like Asia, Mexico and Africa where guava is easily available.^24^,^25^ The guava leaf and fruit are deemed safer, more compatible with biological systems and more effective than their stem, peel, pulp and skin.^28^

Guava displayed enhanced anti-microbial activity against several pathogens, such as *Pseudomonas aeruginosa, Enterobacter cloacae, Staphylococcus aureus, Bacillus, Enterococcus faecalis, Salmonella, Escherichia coli, Shigella flexineri, Klebsiella and Actinomycetes*, in comparison to apple and pomegranate in certain in-vitro experiments.^29^-^31^ The presence of large amounts of methanol in guava extract significantly enhances its antimicrobial potential.^31^ Guava’s convenience, biological security and cost-effectiveness make it an ideal choice for the commercial herbal based oral gel used to treat mouth ulcers in the current study. In developing countries such as Pakistan, cost effectiveness and symptom relief are the primary factors determining medication choice. Further studies are recommended to involve the broader range participants from different areas in order to avoid the demographic and environmental homogeneity for better external validity.

### Limitations:

The study was conducted at one clinical setting (public sector hospital) of Islamabad where participants shared the similar geographical and demographic features. Further investigations are required to be conducted in the large sample size so that the results of the trials can become applicable in the clinical settings.

## CONCLUSION

The current study concluded that *Psidium guajava* based- herbal gel is a natural remedy for quick and efficient treatment of recurrent aphthous stomatitis (oral ulcers) in short time duration. This gel is potent enough to reduce the ulcer size efficaciously and alleviate the symptoms without inducing any adverse effects. Naturally procured oral gels can be considered as a strong, stable and cost-effective solution for the palliative management of minor Recurrent Aphthous Stomatitis (RAS) on regular basis.

## Data Availability Statement:

All data generated or analyzed during the study is available in this manuscript.

### Authors’ Contribution:

**AM:** Conceptualization.

**AM, EM, EZ M and EF M:** Methodology.

**EM, EZ M, EF M:** Data Collection.

**EM, EZ M and EF M:** Data Processing.

**EM, EZ M and EF M:** Writing - Original Draft.

**AM:** Data Analysis & Interpretation, Writing - Review & Editing, Supervision, Critical Review, responsible for integrity of study.

All names seems same. You need to use different abbreviation for the authors.
